# A Claudin-Based Molecular Signature Identifies High-Risk, Chemoresistant Colorectal Cancer Patients

**DOI:** 10.3390/cells10092211

**Published:** 2021-08-26

**Authors:** Saiprasad Gowrikumar, Mark Primeaux, Kristina Pravoverov, Chao Wu, Bryan C. Szeglin, Charles-Etienne Gabriel Sauvé, Ishwor Thapa, Dhundy Bastola, Xi Steven Chen, J. Joshua Smith, Amar B. Singh, Punita Dhawan

**Affiliations:** 1 Department of Biochemistry and Molecular Biology, University of Nebraska Medical Center, Omaha, NE 68198, USA; sai.gowrikumar@unmc.edu (S.G.); mark.primeaux@unmc.edu (M.P.); kristina.pravoverov@unmc.edu (K.P.); amar.singh@unmc.edu (A.B.S.); 2 Department of Surgery, Colorectal Service, Memorial Sloan Kettering Cancer Center, New York, NY 10065, USA; wuc1@mskcc.org (C.W.); bryanszeglin@gmail.com (B.C.S.); sauvec@mskcc.org (C.-E.G.S.); smithj5@mskcc.org (J.J.S.); 3 Human Oncology and Pathogenesis Program, Memorial Sloan Kettering Cancer Center, New York, NY 10065, USA; 4 Albert Einstein College of Medicine, Bronx, NY 10461, USA; 5 College of Information Science & Technology, University of Omaha, Omaha, NE 68182, USA; ithapa@unomaha.edu (I.T.); dkbastola@unomaha.edu (D.B.); 6 Department of Public Health Sciences, Sylvester Comprehensive Cancer Center, University of Miami Miller School of Medicine, Miami, FL 33136, USA; steven.chen@med.miami.edu; 7 VA Nebraska-Western Iowa Health Care System, Omaha, NE 68105, USA; 8 Buffet Cancer Center, University of Nebraska Medical Center, Omaha, NE 68105, USA

**Keywords:** claudin-1, claudin-7, CRC, chemoresistance, metastasis, molecular signature

## Abstract

Identifying molecular characteristics that are associated with aggressive cancer phenotypes through gene expression profiling can help predict treatment responses and clinical outcomes. Claudins are deregulated in colorectal cancer (CRC). In CRC, increased claudin-1 expression results in epithelial-to-mesenchymal transition and metastasis, while claudin-7 functions as a tumor suppressor. In this study, we have developed a molecular signature based on claudin-1 and claudin-7 associated with poor patient survival and chemoresistance. This signature was validated using an integrated approach including publicly available datasets and CRC samples from patients who either responded or did not respond to standard-of-care treatment, CRC cell lines, and patient-derived rectal and colon tumoroids. Transcriptomic analysis from a patient dataset initially yielded 23 genes that were differentially expressed along with higher claudin-1 and decreased claudin-7. From this analysis, we selected a claudins-associated molecular signature including PIK3CA, SLC6A6, TMEM43, and ASAP-1 based on their importance in CRC. The upregulation of these genes and their protein products was validated using multiple CRC patient datasets, in vitro chemoresistant cell lines, and patient-derived tumoroid models. Additionally, blocking these genes improved 5-FU sensitivity in chemoresistant CRC cells. Our findings propose a new claudin-based molecular signature that associates with poor prognosis as well as characteristics of treatment-resistant CRC including chemoresistance, metastasis, and relapse.

## 1. Introduction

Colorectal cancer (CRC) is the second leading cause of cancer-related deaths worldwide, with an approximate 5-year survival rate (all stages included) of 64.4% [1]. The overall risk of mortality due to CRC has been falling due to advancements in early detection and the development of novel therapies. However, CRC incidence is rising in adults under the age of 50, and the survival rate for refractory or metastatic CRC remains dismal [2]. Furthermore, variation in treatment responses has made optimizing patient care strategies particularly challenging, as there is not yet an accurate method for predicting response to chemotherapy or recurrence risks in CRC patients [3]. So far, the treatment decisions rely on TNM histopathologic staging and the presence of high-risk clinical features such as perforation and obstruction [4]. Usually, surgery in combination with 5-fluorouracil (5-FU)-based adjuvant chemotherapy is the standard treatment option for Stage II and Stage III CRC patients, with unfavorable prognosis based on current methods of characterization. However, treatment responses can vary, even among patients with similar clinical or histopathologic features [5]. Therefore, more sensitive, and specific methods should be explored to determine the degree or risk of tumor aggressiveness and chemosensitivity as a complement to preliminary risk assessment based on tumor staging.

It has been reported that characterization of molecular biomarkers associated with aggressive tumor behavior can improve prognostic evaluation and help to identify high-risk patients [6]. Several new biomarkers have been proposed in recent years, including genes involved in cell cycle regulation, apoptosis, metastasis, epithelial-to-mesenchymal transition (EMT), differentiation of cancer stem cells, and angiogenesis [7,8]. There have been many attempts to determine predictive factors for responses to chemotherapy; however, they have not yet been routinely applied in clinical practice [9]. If optimized and utilized, biomarker screening may help to stratify patients by disease risk and help to predict responses to standard-of-care chemotherapy, potentially helping to inform the selection of optimal, personalized chemotherapeutic regimens.

This study aimed to determine a gene signature for risk stratification and prognostic evaluation of chemoresistance based on the expression of claudins, a class of tight-junction proteins that are variably expressed in a cell- and tissue-specific manner [10,11]. Recent studies have revealed that claudin expression is dysregulated in various cancers, and the degree of dysregulation is associated with disease severity [12,13]. For example, claudin-low breast cancers display more aggressive behavior, including high invasive potential, mesenchymal features, and poor differentiation [9]. Another group has also defined a claudin-low molecular subtype of high-grade bladder cancer which also exhibits similar aggressive characteristics [14]. These tumors were found to be enriched for genes associated with EMT, immune cell chemotaxis, and cancer stemness, features that are associated with poor prognosis and resistance to conventional chemotherapeutic agents [14]. These studies have revealed that claudin expression is a useful way to identify certain cancer subtypes with clinically relevant characteristics.

Our group and others have previously demonstrated that claudins are highly dysregulated in colorectal cancer. For example, high expression of claudin-1 (CLDN1) is associated with high-risk tumor behavior [15,16]. CLDN1 increases in a stage-specific manner and correlates with EMT, invasion, metastasis, and poor outcomes in CRC [16]. We have also reported an inverse relationship between CLDN7 expression and CRC progression. CLDN7 decreases in a stage-specific manner in CRC, and its expression can induce mesenchymal to epithelial transition (MET) and suppress tumor formation [17]. Furthermore, CLDN1 and CLDN7 are often inversely associated with one other in CRC [16,17]. Based on these findings, we propose that CLDN1 and CLDN7 may serve as the foundation of a claudin-based gene signature for CRC that can help in the identification of patients who are susceptible to chemoresistant and recurrent tumors.

Using a 250-patient cohort transcriptome analysis, we have developed a CLDN-associated molecular signature, stringently selecting genes whose expression is associated with both high CLDN1 and low CLDN7 expression. To test this signature, we used an integrated approach involving CRC patient datasets and samples from CRC patients who either responded or did not respond to standard-of-care treatment along with ex vivo data from CRC cell lines and patient-derived tumoroids. Based on the results of these validation studies, we propose that this CLDN-based molecular signature may help identify high-risk CRC patients who have an elevated risk of recurrence and resistance to standard therapies, and who may require more aggressive treatment strategies.

## 2. Materials and Methods

### 2.1. Cells and Cell Culture

CRC cell lines were obtained from American Type Culture Collection (ATCC; Manassas, VA, USA) and were maintained in RPMI-1640 or DMEM medium (Hyclone, Marlborough, MA, USA) supplemented with 10% (*v*/*v*) inactivated fetal calf serum, penicillin (100 units/mL), and streptomycin (100 µg/mL). Rectal and Colon Patient-Derived Tumoroid culture: cell derivation and culture as previously described [18].

### 2.2. Generation of Chemoresistant Cell Lines

DLD-1 and HT29 CRC cell lines were used to generate 5-FU-resistant (5-FUR)/Oxaliplatin -resistant (oxaR) cultures through chronic exposure to increasing concentrations of 5-FU/Oxaliplatin, as previously described [19]. Briefly, cells were exposed to increasing levels of 5-FU/Oxaliplatin until able to withstand and proliferate in the presence of a clinically relevant dose of 2 μg/mL [20]. Chemoresistant cell lines were maintained under constant exposure to the drug except during trypsinization and passaging of cells, at which point the drug was added the day after replating.

### 2.3. Colorectal Cancer Tumor Samples

**Figure 1:**
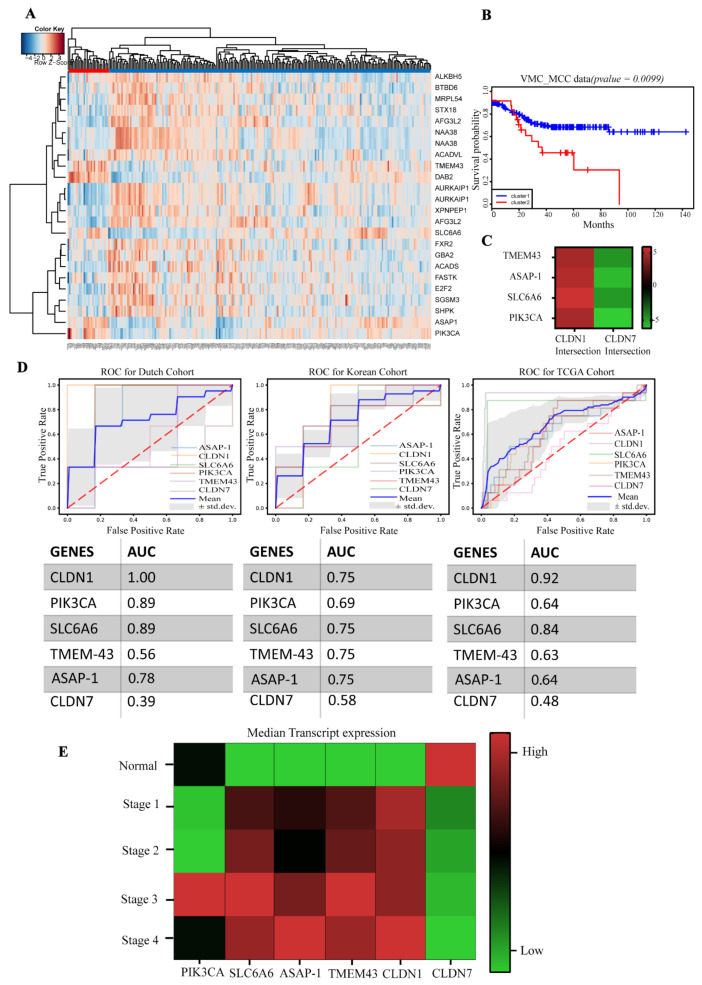
Hierarchical clustering analysis, impact on survival and performance of the genes: (**A**) Unsupervised hierarchal clustering of genes correlated with CLDN1 and CLDN7 as represented by heat map. The color range from blue to red represents the range of gene expression from a downregulated state to an upregulated state. Blue = Downregulated; Red = Upregulated. The horizontal axis represents patients, and the vertical axis represents gene clusters. (**B**) Kaplan–Meier analysis of Cluster 1 and Cluster 2, Cluster 1 (blue) patients demonstrate better overall survival and less recurrent disease than Cluster 2 (*p* = 0.005 and <0.001, respectively). (**C**) The genes associated with increased CLDN1 and decreased CLDN7, which were mostly associated with low relapse-free survival clusters and thus high-risk CRC were filtered for the final gene panel. The expression status of the selected genes is represented as a heat map along with CLDN1 and CLDN7 intersection. The color range from green to red represents the range of gene expression from a downregulated state to an upregulated state. (**D**) The performance of the signature genes was represented using the receiver-operating curve (ROC) curves for the individual genes which were generated based on the cumulative mean AUC. ROC curves were generated with fixed intervals as to average the True and False Positive Rates for multiple ROC curves. The gray region defines +/− one standard deviation from the mean. The AUC values for each gene are represented in the table below. (**E**) The transcriptome analysis from TCGA database between the expressions of PIK3CA, SLC6A6, ASAP-1, TMEM-43, CLDN1, and CLDN7 across CRC samples at different stages compared to normal colon (the data were adopted from UALCAN, and the median value was used to depict the heat map). The median value and respective ± error values are detailed in Supplementary Table S2.

The colon tissue sections from 250 CRC patients who showed responding and non-responding phenotypes to chemotherapeutic treatment were obtained from two different centers (Vanderbilt-Ingram Cancer Center, Nashville, TN, USA and Moffit Cancer Center, Tampa, FL, USA, and the discovery datasets were applied [21]. Note that the discovery dataset has been published to the Gene Expression Omnibus (GEO) database at GSE161158, and the batch effect has been removed using the *ComBat* function from the Bioconductor package *sva* (PMID:166322515). CLDN1 and CLDN7 gene expression were used as the outcomes to identify CLDN1 and CLDN7 associated gene lists based on the above dataset using a regression model in the Bioconductor *LIMMA* package (FDR 0.0001 as the threshold). The interaction of two gene lists contains 24 probes (23 genes), which were applied for unsupervised clustering illustrated in Figure 1A. Kaplan–Meier analysis and log-rank test were used to determine the differences in DFS between the two clusters in Figure 1B.

### 2.4. Xenograft Tumor Samples

The mouse xenografts were generated using SW480^Con^ and SW480^CLDN1^ CRC cells in our previous study, and the tissue sections were used in the current study [16]. The same xenografts were used for orthotopic caecal implantation to generate metastatic lesions in the liver. The tumor nodule and adjacent normal liver tissue were excised and subjected to protein preparation. All animal experiments were conducted with the approval of the Institutional Animal Care and Use Committee (IACUC) of UNMC protocol number 16-021-04-FC.

### 2.5. Determining IC50

The IC50 of 5-FU/Oxaliplatin for the experimental cells was determined as previously described [22]. Cells were seeded in 96-well plate in triplicate with 100 μL culture medium per well containing 10% FBS, followed by incubation at 37 °C and 5% CO_2_; after 24 h, the cells were treated with varying doses of 5-FU (160 µM–0.125 µM). Presto Blue Cell viability reagent (10 μL per well; Invitrogen, Carlsbad, CA, USA) was added, and the cells were incubated for 10 min at 37 °C prior to fluorescence measurement at 560/590 nm using a universal plate reader (Universal Reader Victor; Perkin Elmer Life and Analytical Sciences, Waltham, MA, USA). Cell viability was then compared to untreated controls. IC50 values were calculated using logarithmic regression (R^2^ > 0.9). The concentrations (X) of 5-FU/Oxaliplatin are depicted X = Log(X). Values are presented as the mean ± SD from three replicates.

### 2.6. Real Time PCR

Total RNA was extracted from cells using Qiagen RNA isolation kit (Hilden, Germany). cDNA was synthesized from RNA with iScript™ Reverse Transcription Supermix (Bio-Rad, Hercules, CA, USA) and performed qPCR. The primer pairs are listed in Supplementary Table S1.

### 2.7. Western Blotting

Cells were harvested and rinsed twice with PBS. Total cell extracts were prepared with RIPA lysis buffer and protein concentration was measured using Bradford reagent (Bio-Rad Laboratories, Hercules, CA, USA), and western immunoblotting was performed. The transferred blots were incubated with respective antibodies, the details of the antibodies are listed in the Supplementary Table S1. The signal was visualized with HRP-conjugated secondary antibodies using enhanced chemiluminescence ((Bio-Rad Laboratories, Hercules, CA, USA).

### 2.8. Sphere Formation Assay

The sphere formation assay was performed as previously described [23]. The parental and chemoresistant cells were maintained in sphere-forming medium consisting of DMEM/F12 supplemented with N2 (Invitrogen) and B27 (Invitrogen) supplements, human recombinant epidermal growth factor (20 ng/mL), and basic fibroblastic growth factor (20 ng/mL) (PeproTech Inc., Rocky Hill, NJ, USA). They were seeded at a density of 200 cells/well in 24-well ultra-low attachment plates, and spheres that arose after 2 weeks were recorded and imaged under a light microscope.

### 2.9. Nanog Reporter Assay

Parental and 5-FUR cells were transfected with the pGreenZeo™ Nanog reporter plasmid obtained as a gift from Dr. Justin Lathia (Cleveland Clinic, Cleveland, OH, USA). After 48 h, the GFP fluorescence was imaged.

### 2.10. Immunofluorescence

The formalin-fixed, paraffin-embedded patient tissue sections were processed through hydration using routine immunofluorescence protocol. The tissue sections were incubated with primary antibodies followed by Cya-3 or FITC-conjugated secondary antibodies. The nuclei were visualized using DAPI staining (Vector laboratories, Burlingame, CA, USA). Images were obtained using the LSM 800 Laser Scanning Microscopic system (Carl Zeiss, Oberkochen, Germany).

### 2.11. Validation of the Genes in the Patient Datasets

Multiple cohorts of data were downloaded from NCBI Gene Expression Omnibus (GEO) and the Cancer Genome Atlas (TCGA) repositories. GEO datasets included in this study consisted of two studies labelled as cohorts. The TCGA data set consisted of 647 tumor and 51 normal tissue samples, which were analyzed using the Data-Driven Reference (DDR) pipeline developed by our group. The analysis consisted of gene expression profiling followed by determining the classification efficiency of each signature gene set of interest. To classify normal and tumor samples in each cohort, a machine learning model based on support vector machine was implemented in Python programming language using 70/30 train test split. To achieve the best possible results, we incorporated hyperparameter optimization and oversampling using the Synthetic Minority Oversampling Technique (SMOTE). The ROC curve provides a visual description of the trade-off between false-positive and true-positive rates for all possible threshold values, and the AUC (area under the curve) measure is the most commonly-used index to estimate the global discriminative power of a diagnostic test.

### 2.12. Statistical Analysis

All data are presented as the mean ± standard deviation. GraphPad Prism (San Diego, CA, USA) was used to perform statistical comparisons involving multiple groups. Student’s *t*-test and one-way ANOVA were used to compare differences between groups. Significance was defined as a *p*-value */^#^ *p* < 0.05; **/^##^ *p* < 0.01; ***/^###^ *p* < 0.001; ****/^####^ *p* < 0.0001 versus respective control.

## 3. Results

### 3.1. Identification of a Claudin-Associated Molecular Signature

To identify a claudin-associated molecular signature which can risk-stratify CRC patients, an analysis was performed using a dataset of 250 CRC patients (Stages I-IV, of which a majority (158 patients) were Stage II/III) from Vanderbilt and Moffit cancer centers (VUMC/MCC) [21].

Because CLDN1 and CLDN7 are inversely involved in CRC progression, EMT, and metastasis, we identified a claudin-associated molecular signature of differentially expressed genes correlating with high CLDN1 and low CLDN7 expression from the transcriptome analysis of the above dataset using a regression model in the Bioconductor *LIMMA* package (FDR 0.0001 as the threshold). A total of 23 genes with significant changes were identified and were then subjected to an unsupervised hierarchical cluster analysis. This analysis then separated the patients into two distinct clusters based on the expression patterns of the 23 significant genes (Figure 1A). A Kaplan–Meier analysis was used to determine if patient survival correlated with these clusters. Our analysis using the log-rank test results in the KM plot demonstrated that Cluster 2 gene expression was associated with lower relapse-free survival (hazard ratio 2.092 with 95 CI (1.166, 3.755)) compared to Cluster 1, which had higher relapse-free survival, (*p* < 0.001) (Figure 1B). We then identified genes within the signature that were highly associated with low relapse-free survival clusters and thus high-risk CRC. PIK3CA, SLC6A6, ASAP1 and TMEM-43 were highly upregulated in patients with lower relapse-free survival and downregulated in patients with higher relapse-free survival. Therefore, these markers were selected for the molecular signature in addition to CLDN1 and CLDN7 to predict high-risk CRC patients (Figure 1C and Figure S1D).

### 3.2. Validation of the Gene Signature in the TCGA Dataset

**Figure 2:**
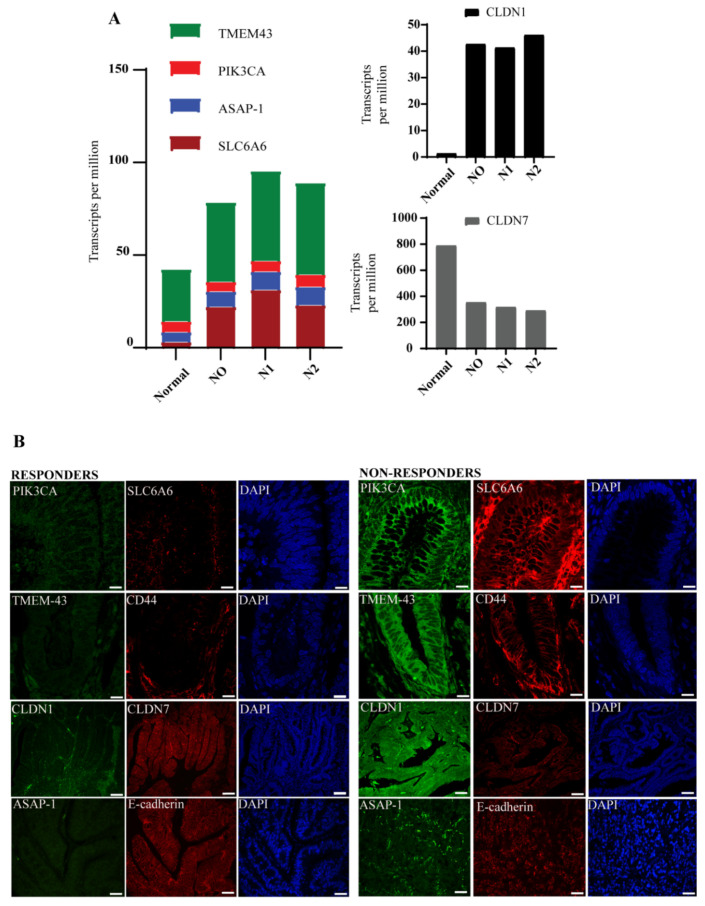
Validation of the genes in responder and non-responder patient samples: (**A**) The transcriptome analysis of PIK3CA, SLC6A6, ASAP-1, TMEM-43, CLDN1, and CLDN7 in nodal colon cancer metastasis datasets from TCGA database through UALCAN; N0- No regional lymph node metastasis, N1- metastasis in 1-3 axillary lymph nodes, N2- metastasis in 4–9 axillary lymph nodes, N3- metastasis in 10 or more axillary lymph nodes. The median value was used to depict the bar graph and the respective ± error values are detailed in Supplementary Table S3. (**B**) The colonic sections from patients who either responded or did not respond to chemotherapy were immunostained with anti-PIK3CA (green), anti-SLC6A6 (red), anti-TMEM-43 (green), anti-CD44 (red), anti-ASAP-1 (green), anti-E-cadherin (red), anti-CLDN1 (green), and anti-CLDN7 (red) antibodies. The sections were counter-stained with the DAPI (blue) nuclear stain (Magnification: 40× Scale bar: 50 µm). The stained sections were visualized under confocal microscope using excitation/emission wavelength for DAPI (529 nm/620 nm), FITC (494 nm/525 nm), and Cya-3 (550 nm/570 nm).

Based on our initial findings in the VUMC/MCC cohort, we hypothesized that this signature would be present in other publicly available CRC datasets. We analyzed three different datasets (Dutch, Korean, TCGA), and the results from the classification step are visually represented by receiver operating characteristic (ROC) curves as shown in Figure 1D. For each cohort, a classifier’s ROC curve was created for each signature gene which represents its true positive rate versus its false positive rate. The average performance for all signature genes is reported for each cohort (shown by the corresponding blue curve in Figure 1D). To examine the expression status of these genes in different stages of CRC, we queried the TCGA database through UALCAN (http://ualcan.path.uab.edu/index.html Dated 7 July 2021), an online database web portal. Expression levels of PIK3CA, SLC6A6, TMEM43, and CLDN1 were significantly elevated in Stage II and III and remained elevated through advanced stages compared to the normal tissue; as expected, this was in contrast to CLDN7 which showed an inverse expression pattern (Figure 1E). In addition, TCGA data showed a clear upregulation of PIK3CA, SLC6A6, ASAP1, TMEM-43, and CLDN1 in the stage-wise advancement of nodal metastasis, with an inverse expression pattern for CLDN7 (Figure 2A).

### 3.3. Molecular Signature Distinguishes Responders from Non-Responder Patients

The claudin-associated molecular signature was then assessed in tumor tissue samples taken from patients who responded (*n* = 8) or did not respond (*n* = 8) to first-line chemotherapy (FOLFOX). Immunofluorescent staining revealed elevated expression of CLDN1, PIK3CA, SLC6A6, TMEM-43, and ASAP-1 and decreased expression of CLDN7 in FOLFOX non-responders relative to responders (Figure 2B). Interestingly, these markers also correlated with the expression of the cancer stem cell marker CD44, which is associated with chemoresistance and decreased E-cadherin, an epithelial marker. Collectively, these data suggest that elevated expression of CLDN1, PIK3CA, SLC6A6, TMEM-43, ASAP-1, and CD44, and decreased expression of CLDN7 in CRC are associated with a poor response to FOLFOX chemotherapy, thus supporting our rationale for utilizing this novel CLDN-associated molecular signature to identify high-risk patients.

### 3.4. CLDN1 and CLDN7 Affect Sensitivity of CRC Cells to 5-FU

**Figure 3:**
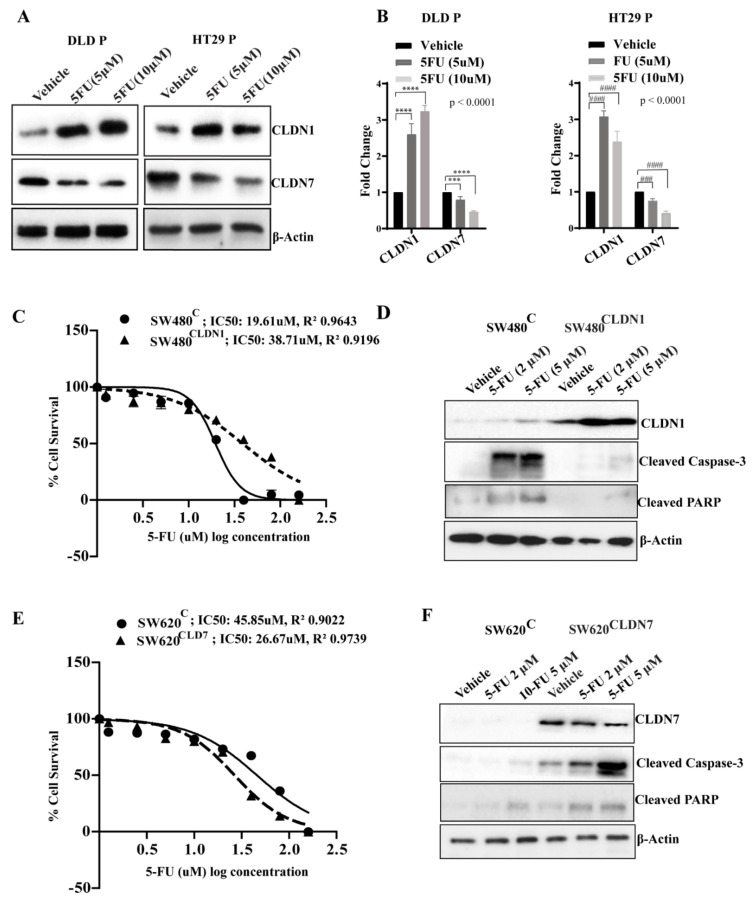
CLDN1 and CLDN7 expression in 5-FU treatment and resistance. (**A**,**B**) Western blot analysis of CLDN1 and CLDN7 in DLD and HT29 cells subjected to 5-FU treatments (5 and 10 µM) and corresponding densitometric analysis of protein levels quantified using ImageJ. β-Actin served as an internal control. The y-axis represents fold change compared to untreated parental (DLD/HT29) cells. Error bars represent the mean ± SD of three independent experiments (*n* = 3). Statistical analysis was conducted using one-way ANOVA with significance represented using the following symbols: ***/^###^ *p* ≤ 0.001,****/^####^ *p* ≤ 0.0001; (**C**) SW480^Con^ and SW480^CLDN1^ cells were treated with a range of 5-FU doses (160 µM to 0.0125 µM); IC50 values were determined using cell viability assay, and the values were fit into a sigmoidal dose-response curve using GraphPad Prism. The concentrations (X) of 5-FU are depicted X = Log(X). Values shown here are representative of 3 independent experiments with 3 replicates. (**D**) Western blot analysis of apoptotic markers cleaved caspase-3 and cleaved PARP in 5-FU treated SW480^Con^ and SW480^CLDN1^ cells. β-Actin served as internal control. (**E**) SW620^Con^ and SW620^CLDN7^ cells were treated with a range of 5-FU doses (160 µM to 0.0125 µM); IC50 values were determined using a cell viability assay, and the values were fit into a sigmoidal dose-response curve using GraphPad Prism. The concentrations (X) of 5-FU are depicted X = Log(X). Values shown here are representative of 3 independent experiments (*n* = 3) with 3 replicates. (**F**) Western blotting analysis of apoptotic markers cleaved caspase-3 and cleaved PARP in 5-FU treated SW620^Con^ and SW620^CLDN7^. β-Actin served as internal control.

To investigate the relationship between CLDN1 and CLDN7 expression and chemotherapeutic exposure, DLD and HT29 colon cancer cells were treated with different concentrations of 5-FU for 24 h. Results showed that 5-FU (5 and 10 µM) treatment elicited an increase in CLDN1 expression and a downregulation of CLDN7 expression in both cell lines (Figure 3A,B). To further test their role in chemoresistance, we determined the IC50 for 5-FU in CLDN1-overexpressing SW480 cells (SW480^CLDN1^) and CLDN7-overexpressing SW620 cells (SW620^CLDN7^) along with their respective control cells. SW480^CLDN1^ cells were more resistant to 5-FU, as evidenced by 2.4-fold increase in IC50 as compared to control cells (Figure 3C). Furthermore, treatment with 5-FU resulted in higher expression of the apoptotic marker cleaved caspase-3 in SW480^Con^ cells compared to SW480^CLDN1^ (Figure 3D), indicating that CLDN1 overexpression protected these cells from 5-FU cytotoxicity. In contrast, SW620^CLDN7^ cells demonstrated increased sensitivity to 5-FU as evidenced by a 1.7-fold lower IC50 and increased expression of cleaved caspase-3 with 5-FU treatment compared to control cells (Figure 3E). Treatment with 5-FU resulted in increased apoptosis under CLDN-7 overexpression condition in SW620 cells (Figure 3F), indicating that CLDN7 overexpression sensitized these cells towards 5-FU. These data indicate that CLDN1 expression is associated with increased resistance to 5-FU, while CLDN7 expression is associated with an increased sensitivity to 5-FU.

### 3.5. Validation of the Claudin-Associated Molecular Signature in CRC Cell Lines

**Figure 4:**
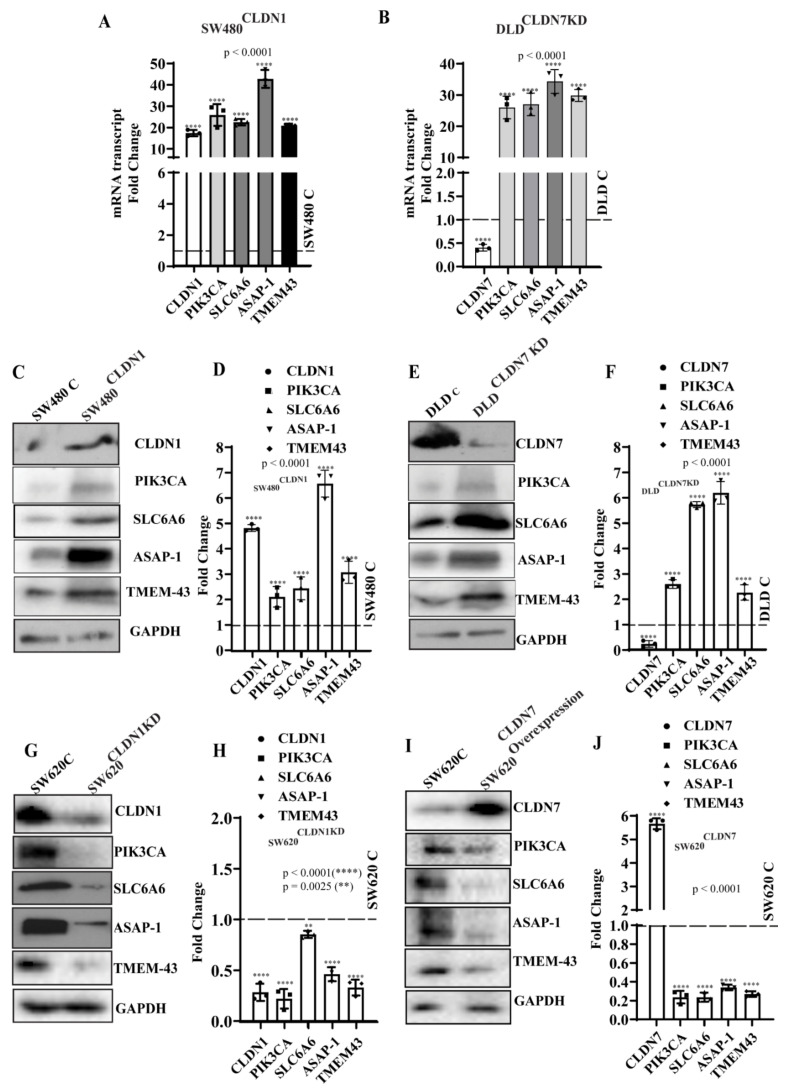
(**A**,**B**) qRT-PCR mRNA transcript analysis of CLDN1, CLDN7, PIK3CA, SLC6A6, ASAP-1, and TMEM-43 in SW480^Con^, SW480^CLDN1^, DLD^Con^, and DLD^CLDN7KD^ cells. Error bars represent the mean ± SD of three independent experiments (*n* = 3). (**C**,**D**) Western blot analysis and the respective densitometries (quantified by ImageJ) of PIK3CA, SLC6A6, TMEM-43, ASAP-1, CLDN1, and CLDN7 in SW480^C^ and SW480^CLDN1^; (**E**,**F**) DLD^Con^, DLD^CLDN7KD^; (**G**,**H**) SW620^C^, SW620^CLDN1KD^; (**I**,**J**) SW620^C^, SW620^CLDN7^ cells. β-Actin served as internal control. Error bars represent the mean ± SD of three independent experiments (*n* = 3). Statistical significance was determined by Student t-test and 1-way ANOVA with post hoc Tukey’s test for pairwise comparison. ** *p* ≤ 0.01, **** *p* ≤ 0.0001.

To test whether PIK3CA, SLC6A6, TMEM-43, and ASAP-1 expression is driven by CLDN1 and CLDN7 expression, we analyzed these genes and proteins in CLDN1-overexpressing SW480 (SW480^CLDN1^) and CLDN7-knockdown DLD-1 (DLD^CLDN7KD^) colon cancer cell lines. These cell lines were chosen because these genetic manipulations result in EMT and tumor progression [16,17]. The transcripts and protein expression for PIK3CA, SLC6A6, ASAP-1, and TMEM43 were increased in both SW480^CLDN1^ and DLD^CLDN7KD^ cells when compared to their respective controls (Figure 4A–F). In contrast, knocking down CLDN1 or overexpressing CLDN7 in SW620 causes a significant decrease in the expressions of PIK3CA, SLC6A6, TMEM-43, and ASAP-1 (Figure 4G–J). These data support the correlation of CLDN1 and CLDN7 with PIK3CA, SLC6A6, ASAP1, and TMEM-43 seen in our database analyses.

To validate the claudin-associated biomarker signature in vivo with respect to an association with CLDN1, we generated xenograft tumors in nude mice from control (SW480^Con^) and CLDN1-overexpressing (SW480^CLDN1^) cell lines. In addition to CLDN1, there was an increase in PIK3CA, SLC6A6, TMEM-43, and ASAP-1 in SW480^CLDN1^ tumors relative to the SW480^Con^ tumors as evidenced through immunostaining (Figure S1A). It was previously shown that SW480^CLDN1^ cells are highly metastatic and metastasize to the liver [16]; similarly, we observed metastatic liver nodules in the SW480^CLDN1^ mice after orthotopic transplantation in the colon. As seen through western blot, these metastatic foci exhibited high expression of CLDN1, PIK3CA, SLC6A6, TMEM-43, and ASAP-1, highlighting the association of CLDN1 with these proteins in the metastatic lesions (Figure S1B).

### 3.6. Signature Gene Expression in Chemoresistant Cell Line Variants

**Figure 5:**
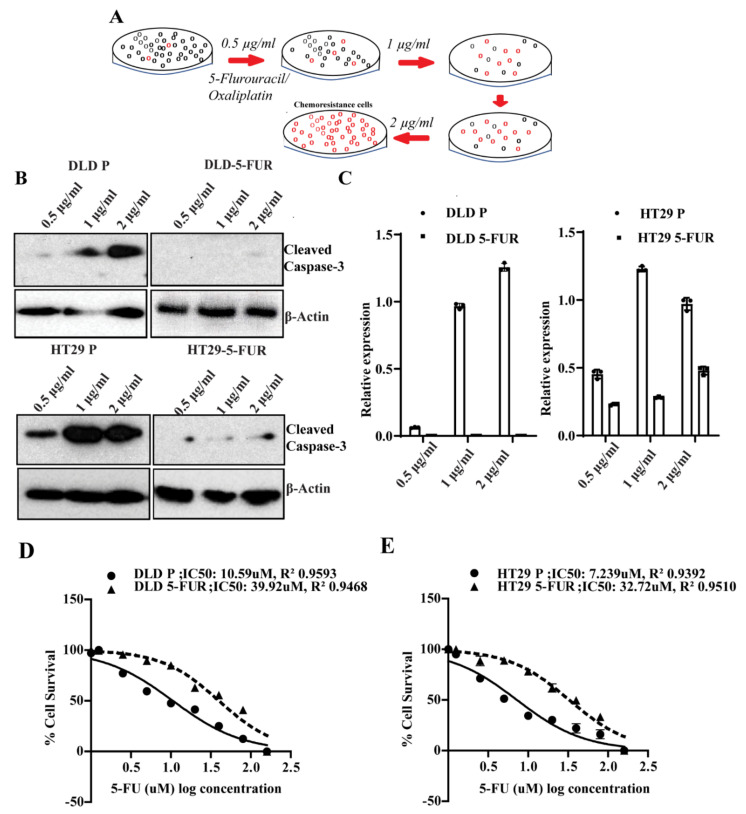
Validation of the signature genes in chemoresistance colon cancer cells: (**A**) Schematic representation of the protocol used to prepare the chemoresistant cells from DLD and HT29 CRC cell lines using incremental doses of 5-FU/Oxa concentration. (**B**) Apoptosis in DLD P/5-FUR and HT29 P/5-FUR cells upon treatment with increasing concentrations of 5-FU as determined by cleaved caspase-3 expression and its corresponding densitometry (**C**) with relative protein expression normalized to reference protein β-Actin used as internal control. (**D**) DLD parental (DLD P)/DLD 5-FUR and (**E**) HT29 parental (HT29 P)/5-FUR cells were treated with the range of 5-FU doses (160 µM to 0.0125 µM); IC50 values were determined using cell viability assay and the values were fit into a sigmoidal dose-response curve using GraphPad Prism. The concentrations (X) of 5-FU are depicted X = Log(X). Values shown here are representative of three independent experiments with three replicates.

**Figure 6:**
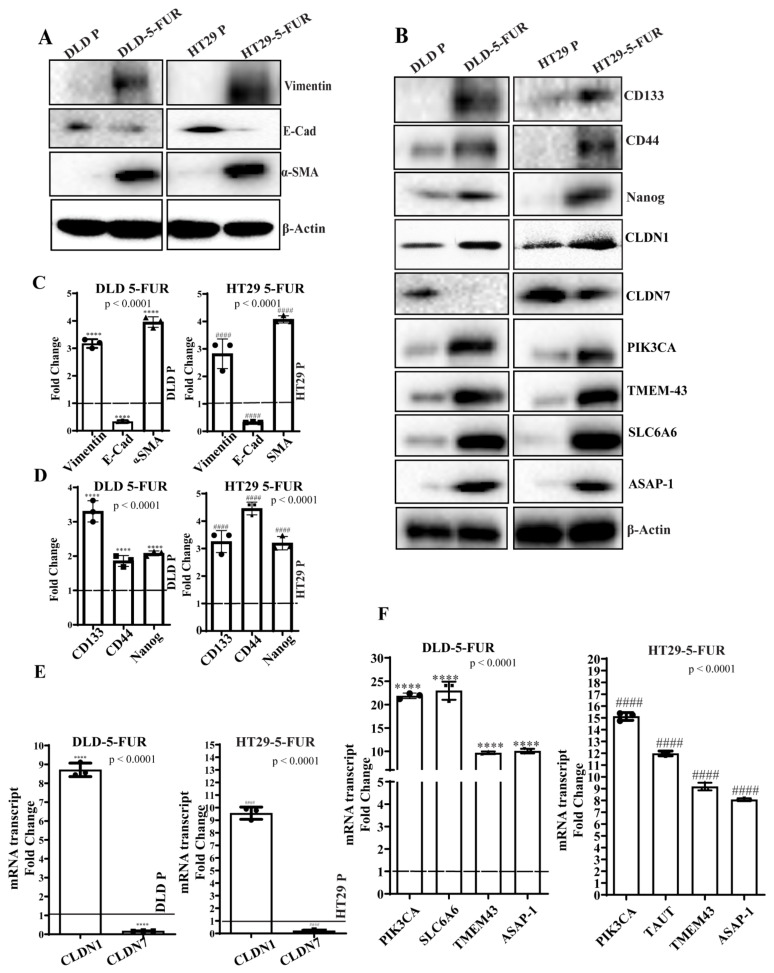
Expression status of markers for EMT and colorectal cancer stem cells along with signature proteins in chemoresistant cells. (**A**) Western blotting analysis of EMT markers Vimentin, E-Cad, α-SMA in DLD and HT29 parental and 5-FUR cells (**B**) Western blot analysis of stem cell markers CD133, CD44, Nanog, CLDN1, CLDN7, PIK3CA, TMEM-43, SLC6A6, ASAP-1, and TMEM-43 in DLD and HT29 parental and 5-FUR cells (**C**,**D**) Densitometry of protein expression for EMT markers Vimentin, E-Cad, α-SMA and stem cell markers CD133, CD44, and Nanog in DLD and HT29 5-FUR cells as normalized against the reference protein β-Actin used as internal control. Y-axis represents fold change compared to DLD/HT29 parental cells. (**E**) mRNA transcript analysis of CLDN1 and 7 in DLD/HT29 parental and 5-FUR cells confirmed through qRT-PCR. (**F**) mRNA transcript analysis of PIK3CA, SLC6A6, ASAP-1 and TMEM-43 in DLD/HT29 parental and 5-FUR cells confirmed through qRT-PCR. Error bars presented are mean ± SD of three independent experiments (*n* = 3). Statistical significance was determined by Student’s t-test and 1-way ANOVA with post hoc Tukey’s test for pairwise comparison. ****/^####^ *p* ≤ 0.0001.

Next, we derived 5-FU chemoresistant (5-FUR) cell line variants of DLD and HT29 CRC cells by culturing these cells in increasing concentrations of 5-FU and selection of surviving cells (Figure 5A). These cells were considered chemoresistant when they were able to stably proliferate in clinically relevant plasma concentrations of 2 μg/mL 5-FU. In addition, cleaved caspase-3 (CC3) expression indicated decreased apoptosis in 5-FUR cells compared to parental cell lines upon 5-FU treatment (Figure 5B,C). Increased resistance was indicated by higher IC50 values for 5-FU when compared to parental cells (Figure 5D,E). It is known that EMT and enrichment of cancer stem cells (CSC) are important phenomena in the development of chemoresistance, and previous studies have demonstrated increased EMT markers and CSC characteristics in chemoresistant cells [24]. To this end, we assessed these properties in the DLD and HT29 5-FUR cells. Mesenchymal markers (α-SMA, vimentin) were upregulated in 5-FUR cells as compared to parental controls, whereas the epithelial marker E-cadherin was down-regulated (Figure 6A). We evaluated stemness through sphere-forming ability and the expression of established CRC stem cell markers in both DLD and HT29 parental and 5-FUR variant cells. Western blot analysis showed an enhancement of the stem cell transcription factor Nanog, along with the colon cancer stem cell markers CD133 and CD44 in 5-FUR variant cells when compared to parental cells (Figure 6B). Immunofluorescent staining also showed a notable increase in CD133 and CD44 in 5-FUR variant cells (Figure S2A). Non-adherent spheres formed in DLD and HT29 5-FUR cells were greater in number and size when compared to corresponding parental cells (Figure S2C). In addition, the transfection of a GFP-Nanog reporter plasmid in DLD and HT29 5-FUR variant cells confirmed the increased expression of GFP-Nanog in 5-FUR cells relative to parental cells (Figure S2D). These data show that resistance to 5-FU is concomitant with an increase in markers for EMT and a more colon cancer stem cell-like phenotype. Next, we assessed the status of claudin-based molecular signature in the 5-FU resistant cells. As evidenced by RT-PCR, Western blotting, and immunofluorescent staining, DLD and HT29 5-FUR variant cells displayed an increase in CLDN1, PIK3CA, SLC6A6, TMEM-43, and ASAP-1 as well as a decrease in CLDN7 at both the transcriptional and translational levels (Figure 6B,E,F and Figure S2E,F). This indicates that the expression of CLDN-based signature proteins is associated with the development of chemoresistance in CRC cells. Given that oxaliplatin is also a standard chemotherapeutic agent used in colon cancer treatment, we also examined the expression of molecular signature genes PIK3CA, SLC6A6, ASAP-1 and TMEM-43 in colon cancer cells resistant to this drug. Using the technique described in Materials and Methods, we developed lines of oxaliplatin-resistant DLD and HT29 cells (DLD-OxaR and HT29-OxaR). Resistance was confirmed in DLD-OxaR and HT29-OxaR by increased IC50 values for oxaliplatin (Figure S3A) and decreased expression of cleaved caspase-3 in response to drug treatment when compared to parental cells (Figure S3B). These cells also display increased markers for EMT and stemness similar to 5-FUR cell lines (Figure S3D). RT-PCR and western blot analysis show and increase in expression of the signature genes at both the transcriptional and translational level (Figure S3F–H). These data show that our molecular signature is applicable to both 5-FU and oxaliplatin resistance.

### 3.7. Knockdown of PIK3CA and SLC6A6 Suppresses Stemness and EMT Markers and Enhances Chemosensitivity in DLD/HT29 5-FUR Cells

**Figure 7:**
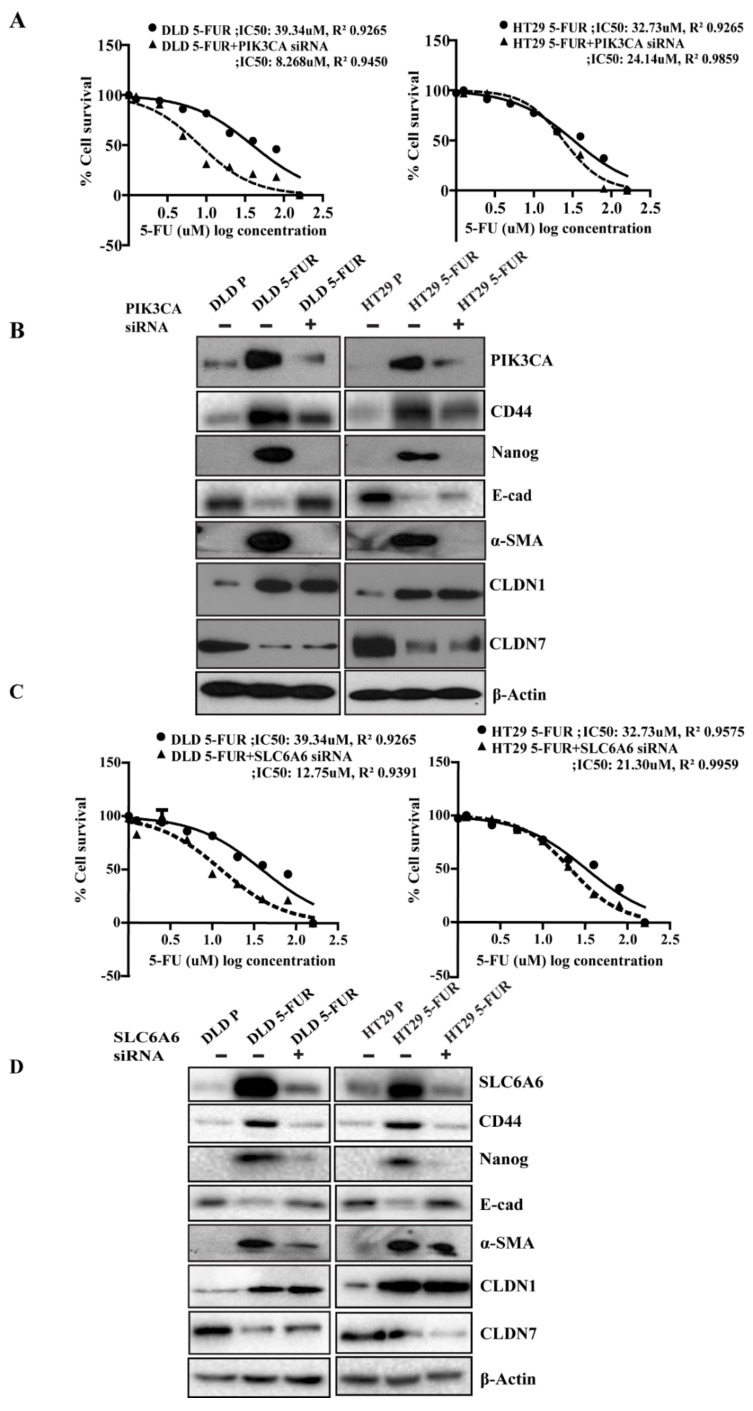
PIK3CA and SLC6A6 silencing decreases resistance to 5-FU in 5-FUR cells: (**A**) The DLD 5-FUR/PIK3CA silenced 5-FUR cells and HT29 5-FUR/PIK3CA silenced 5-FUR cells were treated with the range of 5-FU doses (160 µM to 0.0125 µM); IC50 values were determined using cell viability assay, and the values were fit into a sigmoidal dose-response curve using GraphPad Prism. Values shown here are representative of 3 independent experiments (*n* = 3) with 3 replicates. The concentrations (X) of 5-FU are depicted as X = Log(X). (**B**) Western blot analysis of the cancer stem cell and EMT markers (CD44, Nanog, E-cad, and α-SMA), CLDN1, and CLDN7 in PIK3CA-silenced DLD-FUR/HT29-FUR cells, compared to 5-FUR and parental cells. Densitometry quantified using ImageJ and included in Supplementary Figure S5. (**C**) The DLD 5-FUR/SLC6A6 silenced 5-FUR cells and HT29 5-FUR/PIK3CA silenced 5-FUR cells were treated with the range of 5-FU doses (160 µM to 0.0125 µM); IC50 values were determined using cell viability assay and the values were fit into a sigmoidal dose-response curve using GraphPad Prism. Values shown here are representative of 3 independent experiments (*n* = 3) with 3 replicates. The concentrations (X) of 5-FU are depicted as X = Log(X). (**D**) Western blot analysis of cancer stem cell and EMT markers (CD44, Nanog, E-cad, and α-SMA), CLDN1, and CLDN7 in SLC6A6 silenced DLD 5-FUR/HT29 5-FUR cells compared to 5-FUR and parental cells. Densitometry quantified using ImageJ and included in Supplementary Figure S5.

Next, we wanted to investigate the role of the claudin-associated proteins in promoting chemoresistance in CRC cells. Inhibiting PIK3CA or SLC6A6 through siRNA-mediated knockdown in both DLD and HT29 5-FUR variant cell lines resulted in a 2-4-fold decrease in the IC50 value for 5-FU showing enhanced chemosensitivity (Figure 7A,C). In addition, knockdown of either PIK3CA or SLC6A6 resulted in decreased expression of α-SMA and increased expression of E-cadherin along with decreases in CD44, CD133, and Nanog, indicating a loss in markers for EMT and colon cancer stem cells (Figure 7B,D). This effect was also supported by the reduction in sphere forming ability of DLD and HT29 5-FUR when subjected to PIK3CA silencing (Figure S4C).There was no significant difference in the expressions of CLDN1 or CLDN7 suggesting that these proteins may be upstream regulators of PIK3CA and SLC6A6. Functionally, there was also a reduction in sphere-forming ability showing a loss of stem cell-like properties often associated with chemoresistance (Figure S4). Collectively, these findings suggest that PIK3CA and SLC6A6 may promote the development of chemoresistance, in part through the regulation of EMT and cancer stemness.

### 3.8. Metastatic and Recurrent Colonic Tumoroids Display Increased Claudin-Associated Proteins

**Figure 8:**
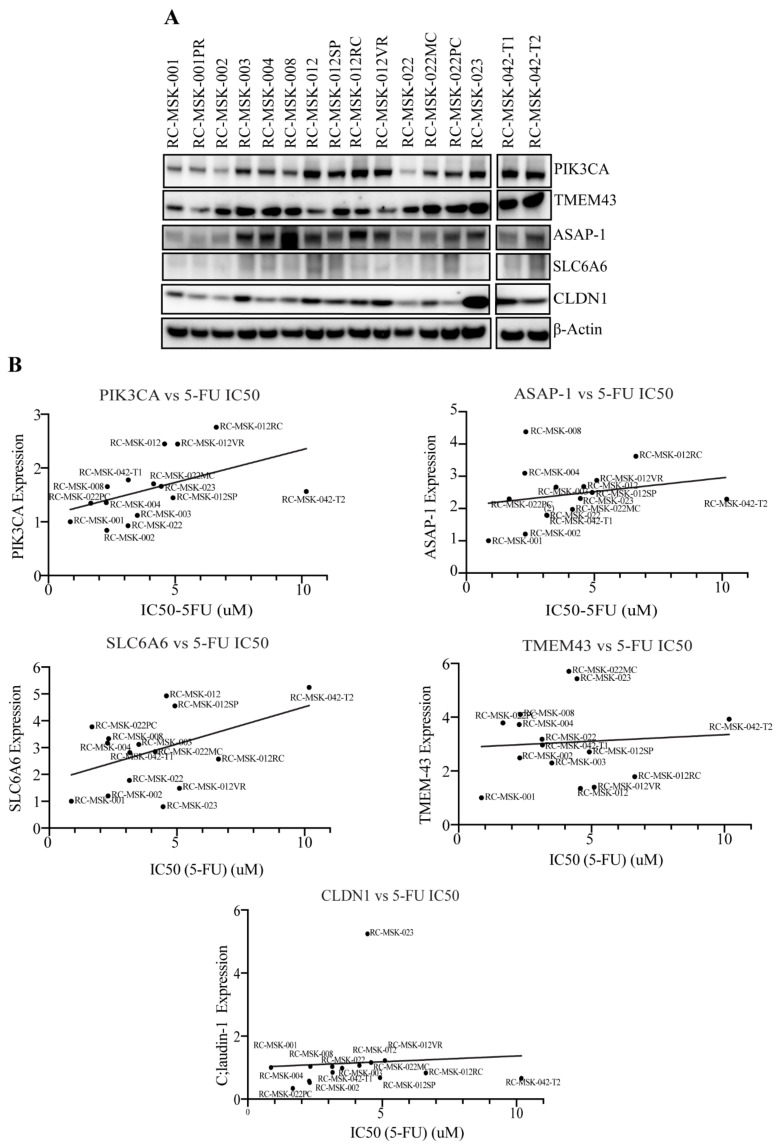
Metastatic rectal and colon tumoroids display elevated expression of Claudin-associated molecular signature proteins with increased tolerance to 5-FU. (**A**) Western blot analysis demonstrates expression of PIK3CA, SLC6A6, TMEM-43, ASAP-1, CLDN1 in the patient-derived tumoroids. PR, perineal recurrence; SP, splenic metastasis; RC, rectal recurrence; VR, vaginal recurrence; MC, mid-chemo; PC, post-chemo; T1 and T2 were sampled from different parts of the same tumor. (**B**) The Pearson’s correlation between the expression of protein and IC50 values displayed by tumoroids is displayed in the box graphs.

To explore the relevance of this signature in patients, we utilized an ex vivo patient-derived rectal and colon cancer organoid platform and analyzed patient-derived tumoroids from a primary rectal and colon (RC-MSK-042) tumor and metastatic sites, including perineal recurrence (PR), splenic metastasis (SP), pelvic recurrence (PRRC), pelvic recurrence in the vagina (PRVG); mid-chemotherapy (MC); and post-chemotherapy (PC). In line with our other observations, CLDN1, CLDN7, PIK3CA, SLCA6A, TMEM-43, and ASAP-1 was expressed in the majority of metastatic tumoroids, irrespective of location, as seen in western blot analysis (Figure 8A). Additionally, there was a positive association between Claudin-associated protein expression and tumoroid IC50 values determined from 5-FU dose-response experiments (Pearson correlation) (Figure 8B).

## 4. Discussion

Current treatment strategies for CRC are far from optimal, mainly due to drug resistance in about half the patients. In both early-stage patients and those with metastatic disease, response to first-line or adjuvant chemotherapy is a strong indicator of overall treatment success [24]. However, there are few methods to predict responses to chemotherapy, so there are few clear recommendations for identifying and appropriately treating high-risk patients who may be prone to early metastasis and/or refractory disease [25]. Major risk assessments for CRC patients are based on tumor stage, grade, and microsatellite instability. While numerous attempts have been made to find multigene signatures associated with CRC prognosis, their utility and accuracy remain uncertain [26,27]. The present study was designed to find a molecular signature associated with the dysregulation of claudins, tight junction proteins known to be associated with high-risk tumors, to aid in the identification of high-risk CRC patients and to help predict response to treatment.

Claudins (CLDNs) are integral tight junction proteins that are aberrantly expressed in a diverse array of human cancers including CRC. The expression profile of claudins changes significantly in many epithelial tumors, and the expression correlates with tumor prognosis [28]. Because of this, CLDNs may serve as effective biomarkers of prognosis and offer potential therapeutic targets [28]. For instance, claudin-low breast cancers which were identified by gene expression profiling are associated with poor survival, high expression of epithelial–mesenchymal transition (EMT) genes, and stem cell-like/less differentiated gene expression patterns [12]. In CRC, experimental data from various studies implicate CLDN1 as a key determinant of invasion and metastasis [29,30]. CLDN1 overexpression also leads to 5-FU and doxorubicin-resistant phenotypes in neoplastic cells [31,32]. In contrast, CLDN7 functions as a potential tumor suppressor and is found to be downregulated in colorectal cancer and metastasis [17]. Studies have demonstrated that CLDN7 is a potential predictive marker for chemosensitivity against cisplatin treatment [33]. Based on this, we have proposed a claudin-associated gene signature for aggressive CRC based on genes that are differentially expressed along with high CLDN1 and low CLDN7.

Our strategy led to the identification of several genes that have previously been linked to aggressive CRC features, including markers of EMT and CSCs. The four genes selected through our process which are dysregulated along with CLDN1 and CLDN7 (PIK3CA, SLC6A6, ASAP1, and TMEM-43) are implicated in a variety of cancers. Upregulation of PIK3CA is frequently observed in CRC, and CRC patients with PIK3CA mutations have poor cancer-specific survival [34]. PIK3CA has also been identified as a key contributor to chemoresistance in metastatic CRC [35]. Furthermore, PIK3CA exon 20 and exon 9 mutations are associated with the sessile-serrated pathway (MSI-H/CIMP-H/BRAF) and serrated pathway (CIMP-L/KRAS (mut)) of CRC tumorigenesis [35]. These features make PIK3CA a promising therapeutic target in CRC. SLC6A6, a sodium and chloride-dependent taurine transporter, is highly expressed in CRC cells compared to normal colonocytes [36]. Moreover, its knockdown attenuated cell survival and enhanced sensitivity to 5-FU treatment while decreasing the frequency of cells with cancer stem cell (CSC)-like properties [36]. ASAP1 is involved in the regulation of cell motility and has been shown to promote cancer cell invasiveness and metastasis, thereby correlating with poor survival in CRC patients [37,38]. Numerous reports have documented the down- or up-regulation of TMEM family proteins in tumor tissues compared to adjoining solid tissues [39,40]. Reports on brain tumors highlighted upregulation of TMEM-43 as a major contributor to poor survival and patient outcomes [41,42]. It is thought that TMEM-43 promotes tumorigenesis and progression via its involvement in EGFR signaling and the activation of NF-κB [42].

Until now, the utility of CLDN1 and CLDN7 dysregulation and their association with PIK3CA, SLC6A6, ASAP-1, and TMEM-43 has not been studied, nor has it been used to predict the risk of recurrence or chemoresistance in CRC patients. Using patient data, we risk-stratified patients based on expression of CLDN1 and CLDN7 and found that the upregulation of CLDN1 and down-regulation of CLDN7 were associated with high-risk patients, and high-risk patients also demonstrated increased expression of PIK3CA, SLC6A6, ASAP-1, and TMEM-43. We confirmed the claudin-associated gene signature in TCGA datasets, tumor samples from patients with tumor recurrence vs. those that responded to therapy, and in patient-derived tumoroids from primary rectal and colon tumors and subsequent metastatic sites. We used statistical measures of discrimination for TCGA dataset. The claudin-associated molecular signature was found to correlate with recurrent (non-responders) and metastatic CRC patients in multiple different models and was also associated with CSC and EMT markers. CSCs play a critical role in CRC metastasis and recurrence and are thereby associated with decreased disease-free survival [43]. Studies have identified a strong relationship between EMT and recurrence rates in Stage II and III CRC [44]. The patient-derived tumoroids from different CRC metastatic sites also support the relationship between the claudin-associated biomarkers and high-risk CRC features. There was robust expression of these genes in rectal and colon tumoroids obtained from different sites of disease including sites of metastases, with claudin-based biomarkers positively correlated with IC50 values for 5-FU sensitivity.

Acquired resistance to chemotherapeutics is a dynamic and multifactorial process that is strongly influenced by EMT and CSC enrichment [45]. The 5-FU resistant DLD and HT29 cell lines further demonstrated upregulation of these biomarkers, along with an increase in CLDN1 and decrease in CLDN7. We observed an enhancement of EMT in both DLD and HT29 5-FUR, as evidenced by increased α-SMA along with a decrease in E-cadherin. It has also been demonstrated that tumors from patients treated with chemotherapy possess a higher frequency of CSCs than tumors from untreated patients [46]. Accumulating evidence suggests an association between the cancer stem cell phenotype and intrinsic chemoresistance [47]. Consistent with earlier reports, our 5-FUR cells displayed an enrichment of CSC markers (CD133, CD44, and Nanog) and sphere-forming ability consistent with the CSC phenotype [48]. The molecular signature proteins PIK3CA, SLC6A6, ASAP-1, and TMEM-43 were clearly upregulated in 5-FUR cells along with both EMT and CSC markers, supporting their association with chemoresistance in CRC.

To ascertain the role of the claudin-associated molecular signature in driving chemoresistance, siRNA-mediated knockdown of PIK3CA or SLC6A6 genes was performed in DLD and HT29 5-FUR cells. Loss of both PIK3CA and SLC6A6 conferred greater sensitivity to 5-FU compared to controls and decreased the frequency of CSC markers. This agrees with previous studies which report the importance of these genes in promoting chemoresistance and metastasis in various cancers [49,50]. The mechanisms of chemoresistance exhibited by cancer cells vary depending on the chemotherapeutic agent in question. To test the validity and applicability of the claudin-associated molecular signature for other drugs such as oxaliplatin, we determined the expression of signature proteins in Oxaliplatin-resistant cells. Oxaliplatin is a third-generation platinum-based compound which also has problems with acquired resistance similar to 5-FU [51]. The performance of the signature was similar to that seen in 5-FU resistance, suggesting the signature’s utility in predicting resistance to other chemotherapeutic agents, as well. 

Our previously published data along with data in this manuscript suggest a mutual interdependence of claudin-1 and claudin-7 expressions. We have previously demonstrated that claudin-1 mediates its pro-carcinogenic and metastatic effects through the activation of Src and Akt signaling, and claudin-7 regulates ERK signaling to modulate EMT in colon cancer [17,52,53]. These pathways are known to regulate PIK3C, SLC6A6, and ASAP-1, while less is known about the regulation of TMEM-43 [53,54,55]. Future studies are needed to investigate the role of these signaling pathways in claudin-1 and 7’s regulation of PIK3CA, SLC6A6, ASAP-1, and TMEM-43.

Overall, this study provides new insights into the risk analysis and treatment of colorectal cancer. We have identified a molecular signature associated with chemoresistance and have validated it through multiple lines of evidence. Future studies will continue to investigate the applicability of our claudin-associated molecular signature to a wider range of chemotherapeutics. Elucidating the molecular pathways which integrate the individual components of this molecular signature could ultimately lead to a better understanding of the mechanisms involved in the development of aggressive and chemoresistant cancer phenotypes.

## Supplementary Materials

The following are available online at https://www.mdpi.com/article/10.3390/cells10092211/s1. The following are available online Figure S1: The expression of the signature proteins in xenograft tumors and metastasis liver tumor’s generated using C and CLDN1 overexpressed cells, Figure S2: The expression of the cancer stem cell markers and the signature proteins in chemoresistance colon cancer cells, Figure S3: The status of signature proteins in Oxaliplatin resistance colon cancer cells and Figure S4: The densitometry analysis of the cancer stem cell markers in PIK3CA and SLC6A6 silenced 5-FUR cells; Table S1: List of antibodies and primers implied in the study, Table S2: The median values of the expressions of TMEM43, SLC6A6, ASAP-1, PIK3CA, CLDN1 and 7 with the +/− error values in the normal and stage wise progressed colon cancer patients adopted from TCGA database, UALCAN. Table S3: The median values of the expressions of TMEM43, SLC6A6, ASAP-1, PIK3CA, CLDN1 and 7 with the +/− error values in the colon cancer nodal metastasis patients adopted from TCGA database, UALCAN. Supplementary methods.
