# Supplementary material for: A Claudin-Based Molecular Signature Identifies High-Risk, Chemoresistant Colorectal Cancer Patients
Source: Cells. 2021 Aug 26;10(9):2211. doi: 10.3390/cells10092211 (PMC8466455; doi:10.3390/cells10092211)
Supplement: Supplementary file 1 [file cells-10-02211-s001.zip › cells-1332738-supplementary.pdf]

cells

MDPI

Article

A Claudin-Based Molecular Signature Identifies High-Risk, Chemoresistant Colorectal Cancer Patients

Saiprasad Gowrikumar [1], Mark Primeaux [1], Kristina Pravoverov [1], Chao Wu [2,3], Bryan C. Szeglin [2,4], Charles-Etienne Gabriel Sauvé [2], Ishwor Thapa [5], Dhundy Bastola [5], X. Steven Chen [6], J. Joshua Smith [2,3], Amar B. Singh [1,7,8] and Punita Dhawan [1,7,8,*]

Supplementary Methods

Antibodies, siRNA, and Reagents

The primary antibodies anti-Claudin-1, anti-Claudin-7, anti-PIK3CA, anti-SLC6A6, anti-TMEM-43, and anti-ASAP-1 were from Invitrogen (Carlsbad, CA, USA). Anti-E-Cadherin was from BD Biosciences (San Jose, CA, USA) and anti-C-Myc was from Santa Cruz Biotechnology (Dallas, TX, USA). Anti-Vimentin, α-SMA, anti-CD133, anti-CD44, anti-Nanog, anti-BCL-xl were from Cell Signaling Technology (Danvers, MA, USA). Antibodies and dilutions are listed in Table 1. Secondary HRP-conjugated antibodies including goat anti-rabbit IgG and goat anti-mouse IgG were from R&D Systems (Minneapolis, MN, USA). The immunofluorescent-tagged secondary antibodies anti-rabbit FITC and anti-mouse Cya3 were from Jackson Immune Research (West Grove, PA, USA). Jet Prime transfection reagent was from Polyplus (New York, NY, USA). 5-FU and Oxaliplatin was obtained from the clinical pharmacy at Nebraska Medicine (Omaha, NE, USA). PIK3CA and SLC6A6 expression were transiently downregulated using two predesigned Silencer Select siRNAs directed against PIK3CA and SLC6A6, and a non-targeting siRNA was used as a negative control (Thermo Fisher Scientific, Waltham, MA, USA).

Cell Transfection

Cells in the logarithmic growth stage were seeded on culture dishes. Upon reaching 70% confluence, SW480 cells were transfected with PCMV script control and claudin-1(CLDN1) overexpression plasmids and were processed for stable preparation. DLD cells were transfected with claudin-7(CLDN7) shRNA plasmid and processed for stable preparation. SW620 cells were transfected with CLDN1 shRNA (Dharmacon) for the stable knockdown of CLDN1. CLDN7 was stably overexpressed using mcherry-claudin-7 construct. DLD 5-FUR and HT29 5-FUR cells were treated with PIK3CA siRNA / SLC6A6 siRNA or control scrambled siRNA for transient knockdown of gene expression. Expression of PIK3CA/SLC6A6 was assessed by Western blot analysis.

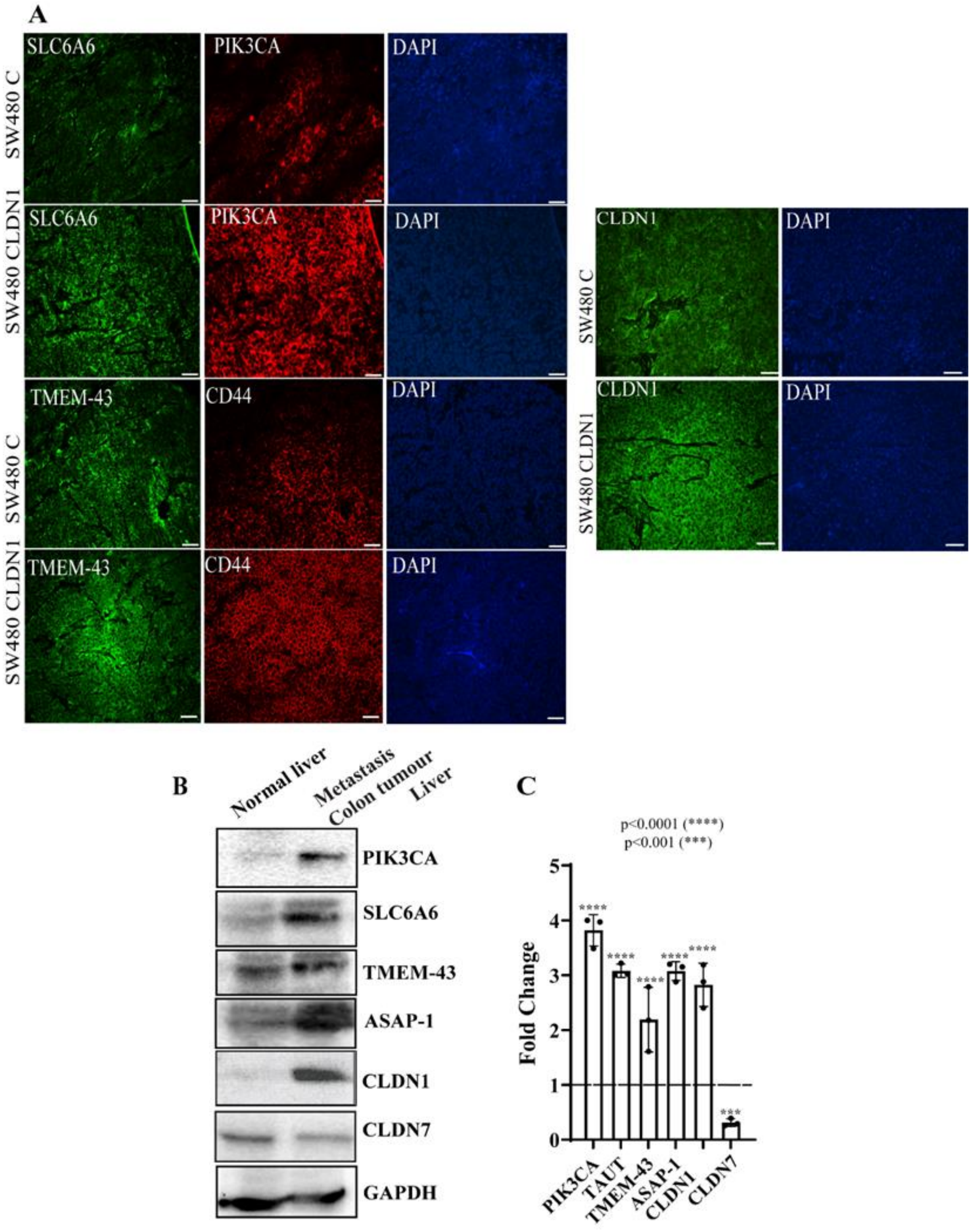

D

GENE SYMBOL	FOLD CHANGE (CLDN1) INTERSECTION)	RAW P VALUE	FOLD CHANGE (CLDN7) INTERSECTION)	RAW P VALUE
TMEM43	4.773527281	3.08E-06	-4.53594	8.89E-06
ASAP-1	5.14679568	5.35E-07	-5.66821	3.95E-08
SLC6A6	5.93989416	9.47E-09	-4.64545	5.48E-06
PIK3CA	4.819917217	2.49E-06	-6.22381	2.02E-09

Figure S1. The expression of the signature proteins in xenograft tumors and metastasis liver tumor's generated using C and CLDN1 overexpressed cells. (**A**) Mouse xenograft tumor sections obtained from nude mice subcutaneously injected with SW480^{Con} and SW480^{CLDN1} were immunostained with anti-SLC6A6, anti-PIK3CA, anti-TMEM-43, anti-CD44, anti-CLDN1 antibodies. Tissue sections were counter stained using DAPI (Blue) to stain nucleus (Magnification: 63, Scale bar: 50 µm). Cells were visualized under confocal microscope using excitation/emission wavelength for DAPI – 529 nm/620 nm, FITC – 494 nm/525 nm and Cya-3 – 550 nm/570 nm emission. (**B**) Western blot analysis of molecular signature proteins PIK3CA, SLC6A6, TMEM-43, ASAP-1, CLDN1 and 7 in normal and metastatic CRC liver lesions emerged through caecal transplantation of xenograft tumors obtained from SW480^{CLDN1} cells in nude mice. (**C**) The relative protein expressions are

normalized to reference protein, β-Actin, used as internal control and its corresponding densitometry quantified using Image J. Y-axis represents fold change compared to normal liver sections. Error bars presented are mean ± SD of three independent experiments (n = 3). Statistical significance was determined by student t test and 1-way ANOVA and post hoc Tukey's test for pairwise comparison. Values sharing following symbol differ significantly, **/## $P \leq 0.01$, ***/### $P \leq 0.001$, ****/#### $P \leq 0.0001$.* – Vs normal liver sections. (**D**) The scores of the differentially expressed genes (TMEM43, ASAP-1, SLC6A6 and PIK3CA) at the Claudin-1 and Claudin-7 intersection with the raw p values.

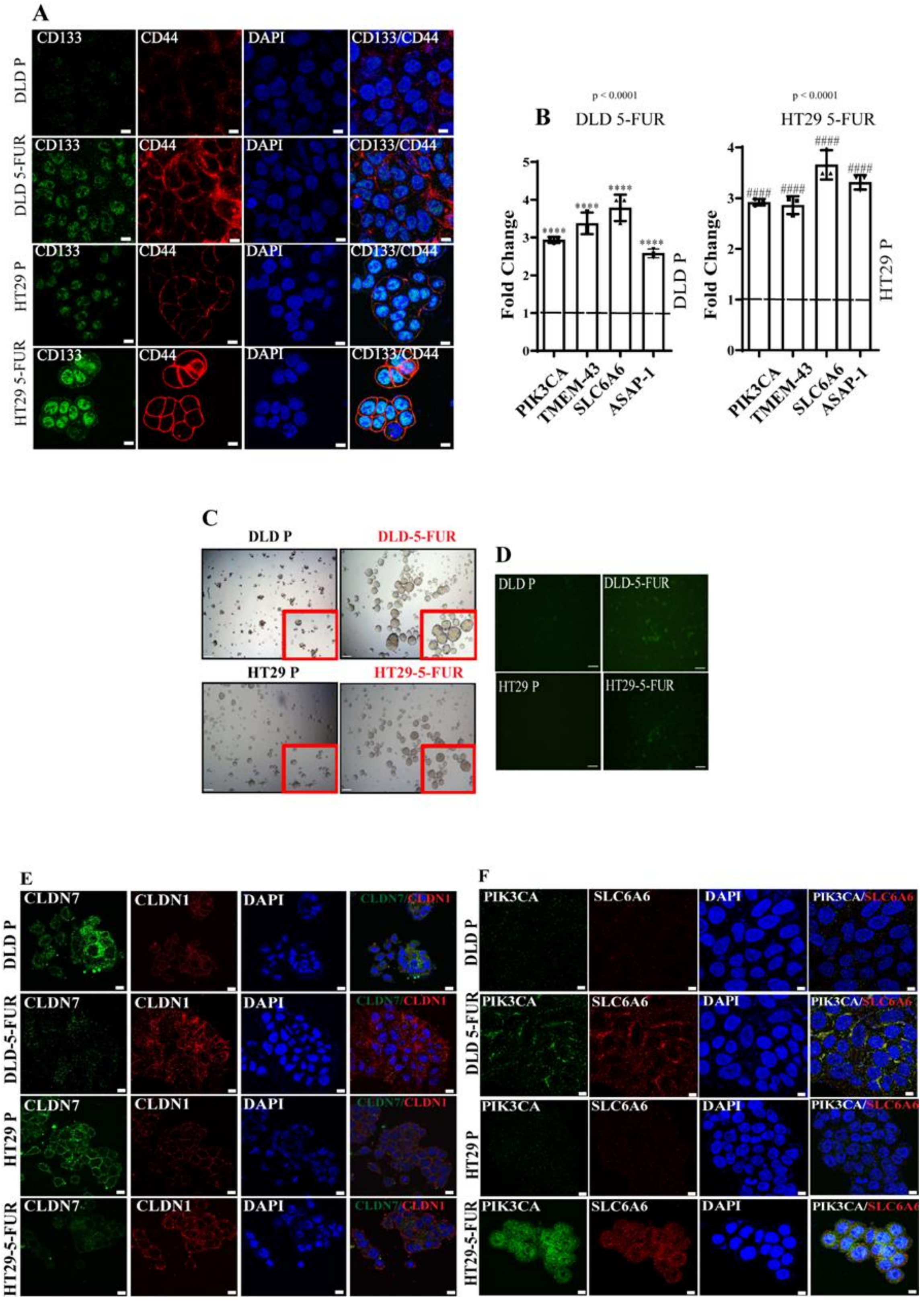

Figure S2. The expression of the cancer stem cell markers and the signature proteins in chemoresistance colon cancer cells. (**A**) The representative images of the DLD and HT29 P/5-FUR cells immunostained with anti-CD133 (green) and anti-CD44 (red). Cells were counter-stained using DAPI (blue) nuclear stain (magnification: 63X, scale bar: 50µm). The stained cells were visualized under confocal microscope using excitation/emission wavelength for DAPI – 529 nm/620 nm, FITC – 494 nm/525 nm and Cya-3 – 550 nm/570 nm emission. (**B**) Densitometry of the protein expressions of PIK3CA, SLC6A6, ASAP-1 and TMEM-43 in DLD and HT29 5-FUR cells as normalized against the reference protein, β-Actin used as internal control. Error bars presented are mean ± SD of three independent experiments (n = 3). Statistical significance was determined by student t test and 1-way ANOVA and post hoc Tukey's test for pairwise comparison. **/## P ≤ 0.01, ***/### P ≤ 0.001, ****/#### P ≤ 0.0001. Values sharing following symbol differ significantly, * – Vs respective un-treated control cells (DLD parental cells); # - Vs respective un-treated control cells (HT29 parental cells). Error bars presented are mean ± SD of three independent experiments (n = 3). Statistical significance was determined by student t test and 1-way ANOVA and post hoc Tukey's test for pairwise comparison. **/## P ≤ 0.01, ***/### P ≤ 0.001, ****/#### P ≤ 0.0001. Values sharing following symbol differ significantly, * – Vs respective un-treated control cells (DLD parental cells); # - Vs respective un-treated control cells (HT29 parental cells). (**C**) Sphere formation assay for DLD and HT29 5-FUR cells compared with respective parental cells, Scale bar-100uM. (**D**) The Nanog reporter plasmid expressions (GFP) in DLD and HT29 5-FUR cells and their respective parental cells as observed through florescence microscope. (**E** and **F**) DLD and HT29 P/5-FUR cells were immunostained with (B) anti CLDN7 (green) and anti-CLDN1 (red), (C) anti-PIK3CA (green), anti-SLC6A6 (red), the representative images are displayed. Cells were counter-stained using DAPI (blue) nuclear stain (magnification: 63X, scale bar: 50µm). The stained cells were visualized under confocal microscope using excitation/emission wavelength for DAPI – 529 nm/620 nm, FITC – 494 nm/525 nm and Cya-3 – 550 nm/570 nm emission.

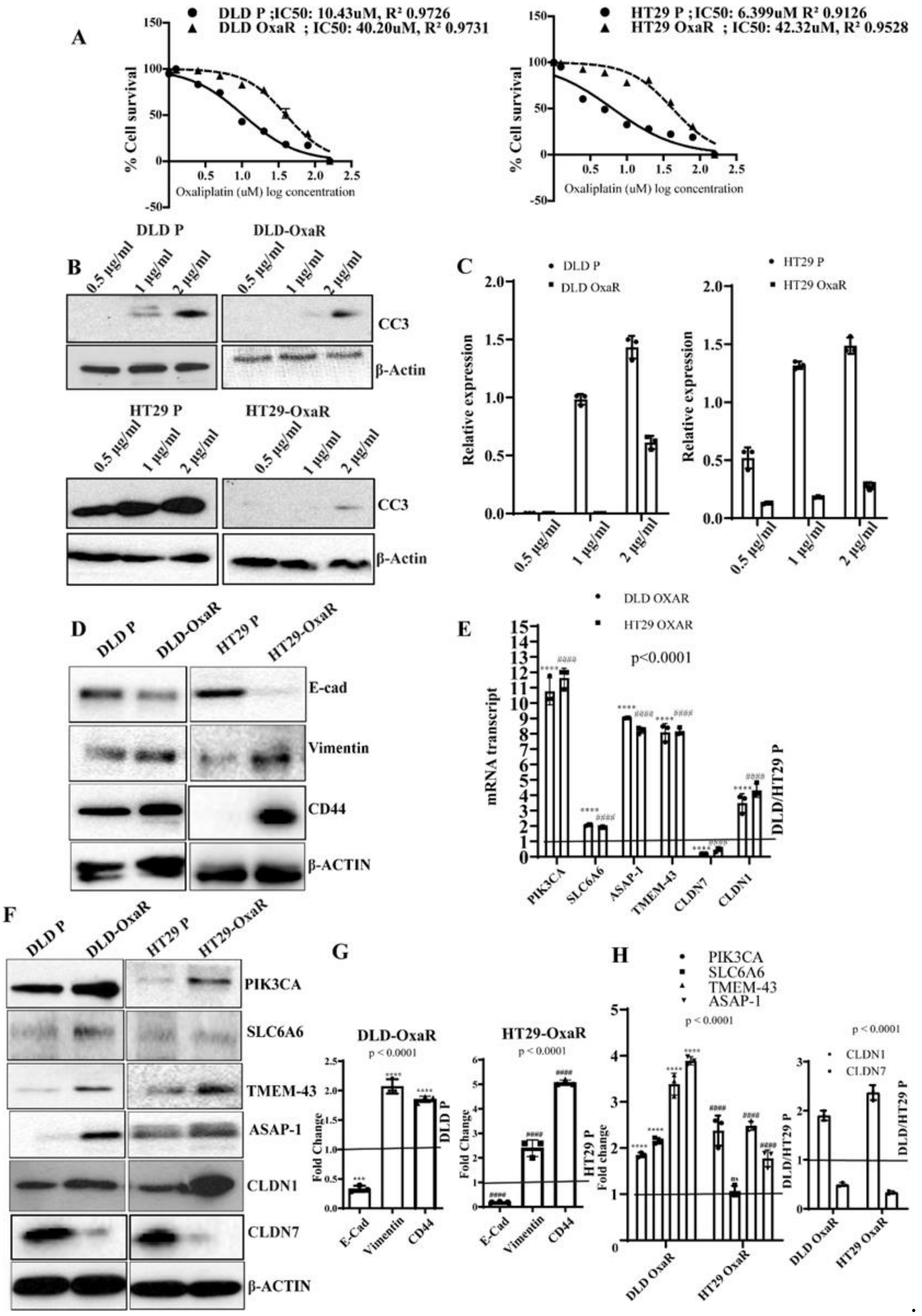

Figure S3. The status of signature proteins in Oxaliplatin resistance colon cancer cells. (**A**) The IC50 Values of the DLD parental/OxaR and HT29 parental/OxaR determined using MTT assay with varying concentration of Oxaliplatin (160 µM to 0.0125 µM), IC50 values were determined using MTT assay and the values were fit into a sigmoidal dose-response curve using Graph Pad Prism Values shown here are representative of 3 independent experiments with 3 replicates. (**B**) The apoptosis intensity in DLD P/OxaR and HT29 P/OxaR cells upon treating with incremental concentration of Oxaliplatin as determined by cleaved caspase-3 expression and its corresponding densitometry (**C**) the relative protein expressions are normalized with reference protein β-Actin used as internal control. Y-axis represents relative expression. (**D**) Western blotting analysis of E-Cad, Vimentin, CD44 in DLD parental/OxaR and HT29 parental/OxaR cells and its respective densitometry, quantified using Image J (**G**); the relative protein expressions are normalized with reference protein, β-Actin

used as internal control, y-axis represents fold change compared to controls. (**E**) The bar graph illustrates the mRNA transcript analysis of PIK3CA, SLC6A6, TMEM-43, ASAP-,1 CLDN1 and 7 in DLD parental/OxaR and HT29 parental/OxaR cells confirmed by qRT-PCR. (**F**) Western blotting analysis of PIK3CA, SLC6A6, TMEM-43, ASAP-1, in DLD parental/OxaR and HT29 parental/OxaR cells and its respective densitometry (**H**). The relative protein expressions are normalized with reference protein, β-Actin used as internal control, y-axis represents fold change compared to controls. Error bars presented are mean ± SD of three independent experiments (n = 3). Statistical significance was determined by student t test and 1-way ANOVA and post hoc Tukey's test for pairwise comparison. $^{**/\#\#}$ $P \leq 0.01$, $^{***/\#\#\#}$ $P \leq 0.001$, $^{****/\#\#\#\#}$ $P \leq 0.0001$. Values sharing following symbol differ significantly, * – Vs respective un-treated control cells (DLD parental cells); $^{\#}$ - Vs respective un-treated control cells (HT29 parental cells).

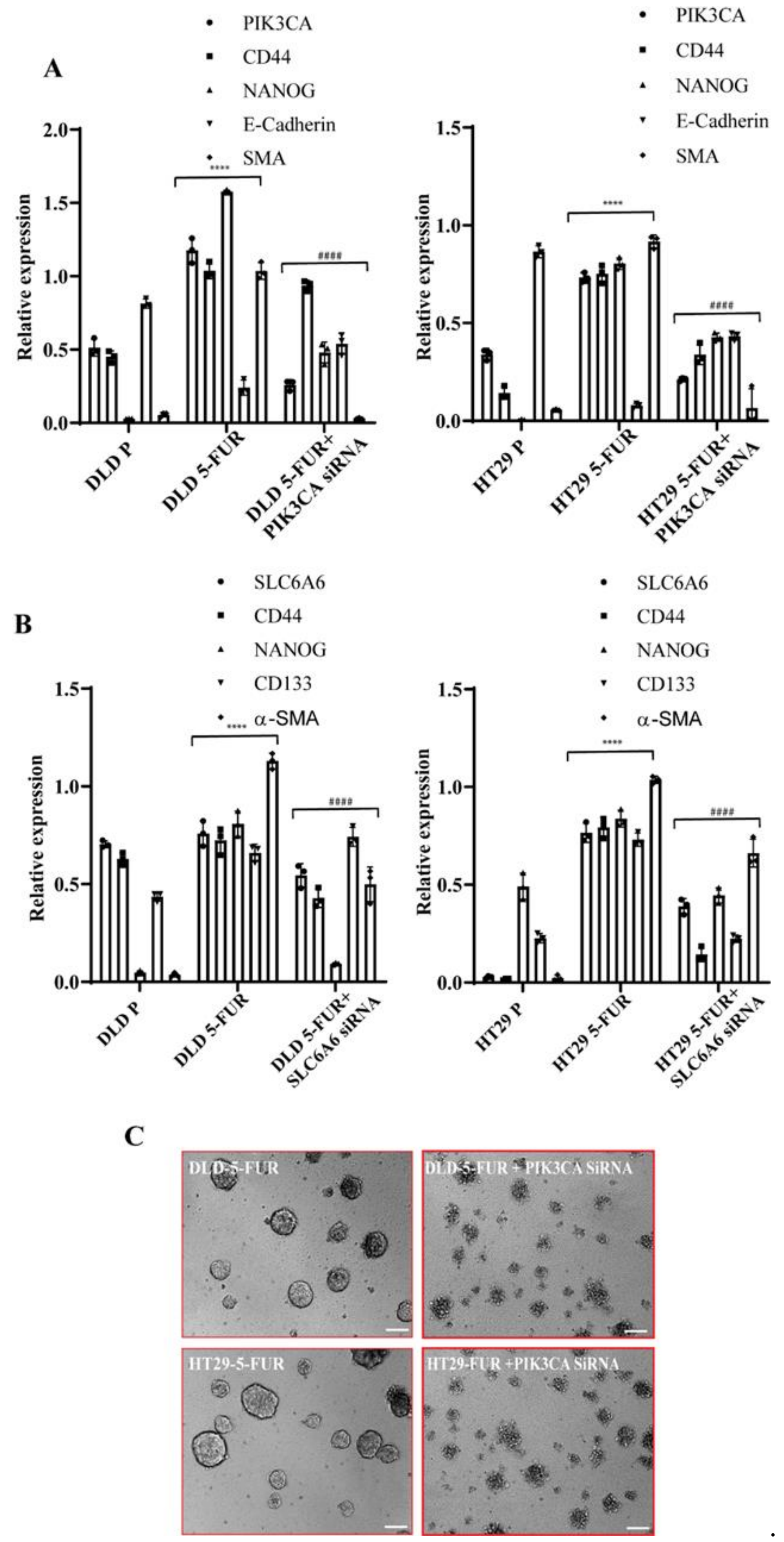

Figure S4. The densitometry analysis of the cancer stem cell markers in PIK3CA and SLC6A6 silenced 5-FUR cells. (**A** and **B**) The relative protein expressions of PIK3CA/SLC6A6, CD44, Nanog, E-cadherin, α-SMA in DLD/HT29 P, PIK3CA-silenced DLD-FUR/HT29-FUR, SLC6A6-silenced

DLD-FUR/HT29-FUR cells normalized against reference protein β-Actin, used as an internal control. Error bars presented are mean ± SD of three independent experiments (n = 3). Statistical significance was determined by student t test and 1-way ANOVA and post hoc Tukey's test for pairwise comparison. **/## $P \leq 0.01$, ***/### $P \leq 0.001$, ****/#### $P \leq 0.0001$. Values sharing following symbol differ significantly, * – Vs respective control cells (DLD/HT29 parental cells); # - Vs respective DLD/HT29 FUR cells. (**B**) The sphere forming ability of DLD/HT29 FUR, PIK3CA-silenced DLD-FUR/HT29-FUR cells analyzed by sphere forming assay. (C) Sphere forming ability of the DLD and HT29 5-FUR cells with and without PIK3CA siRNA.

Table 1. List of antibodies and primers implied in the study.

List of antibodies

**Scheme .**	Antibodies	Dilution	Company	Catalogue number
1.	Claudin-1	1:1000(WB)	Invitrogen	51-9000
2.	Claudin-1	1:100 (IF)	Invitrogen	71-7800
3.	Claudin-7	1:1000(WB)	Invitrogen	34-9100
4.	Claudin-7	1:100(IF)	Invitrogen	34-9100
5.	PIK3CA	1:250 (IF), 1:1000(WB)	Invitrogen	PA5-99395
6.	SLC6A6 (TAUT)	1:100(IF), 1:1000(WB)	Invitrogen	PA5-98161
7.	SLC6A6 (TAUT)	1:250(IF), 1:1000	Invitrogen	PA5-77455
8.	TMEM-43	1:250(IF), 1:1000 (WB)	Invitrogen	PA5-18409
9.	ASAP-1	1:1000(IF), 1:2500(WB)	Invitrogen	PA1-25512
10.	E2F2	1:250(IF), 1:1000(WB)	Invitrogen	PA5-41473
10.	E-Cadherin	1:1000	BD Biosciences Transduction Laboratories.	610182
11.	C-Myc	1:500	Santa Cruz Biotechnology Inc.	SC-40
12.	Vimentin	1:500	Proteintech	10366-1-AP
13.	BCL-xl	1:500	Cell signaling	2764
14.	CD133	1:500	Invitrogen	14-1331-82
15.	CD44	1:500	Cell signaling	3570
16.	Nanog	1:500	Cell signaling	4903

List of primers

S.No	Forward	Reverse
PIK3CA	5′-AAATGAAAGCTCACTCTGGAATTCC-3'	5′-TGTGCAATTCCTATGCAATCG-3′
ASAP1	5′-CCCCTTTTGCAGCAACTTACA-3’	5′-TCCTATGTCCCACAGTAAGCTGG-3’
E2F2	5′-GCCTATGTGACTTACCAGGATATCC-3’	5′-CCTTGACGGCAATCACTGTCT-3’
SLC6A6	5′-TCGACTTTGTGCTGTCTG-3’	5′-GTAGTGGACGACCCTCTT-3’
TMEM-43	5′-TGGCCGCGAATTATTCCAGT-3’	5′-CGTAAGGCGCCAATGATGTG-3’
Claudin-1	5′-CTGGGAGGTGCCCTACTTTG-3’	5′-ACACGTAGTCTTTCCCGCTG-3’
Claudin-7	5′-CAGATGAGCTCCTATGCGGG-3’	5′-CTGATGGCCATACCAGGAGC-3’

Table 2. The median values of the expressions of TMEM43, SLC6A6, ASAP-1, PIK3CA, CLDN1 and 7 with the +/- error values in the normal and stage wise progressed colon cancer patients adopted from TCGA database, UALCAN.

The median values for the transcript of signature genes in different stages of CRC.

**PIK3CA.**	**Median**	**+Error**	**-Error**
Normal (n=41)	5.79	7.77	3.97
Stage 1 (n=45)	5.23	10.38	2.3
Stage 2 (n=110)	5.2	10.59	1.44
Stage 3 (n=80)	6.46	12.5	2.48

Stage 4 (n=39)	5.79	11.35	2.74
**SLC6A6**	**Median**	**+Error**	**-Error**
Normal (n=41)	3.18	5.43	1.85
Stage 1 (n=45)	21.24	99.83	4.56
Stage 2 (n=110)	24.28	97.25	4.3
Stage 3 (n=80)	30.04	91.95	4.67
Stage 4 (n=39)	26.46	88.58	7.92
**ASAP-1**	**Median**	**+Error**	**-Error**
Normal (n=41)	5.38	8.98	2.1
Stage 1 (n=45)	8.8	23.84	2.07
Stage 2 (n=110)	8.23	22.45	2.37
Stage 3 (n=80)	9.24	23.74	2.85
Stage 4 (n=39)	11.17	28.82	3.74
**TMEM43**	**Median**	**+Error**	**-Error**
Normal (n= 41)	28.43	51.68	18.25
Stage 1 (n=45)	42.39	72.67	18.08
Stage 2 (n=110)	43.61	84.42	18.41
Stage 3 (n=80)	48.64	76.95	22.37
Stage 4 (n=39)	46.13	79.94	21.35
**CLDN1**	**Median**	**+Error**	**-Error**
Normal (n= 41)	1.37	3.24	0.22
Stage 1 (n=45)	44.51	151.71	1.97
Stage 2 (n=110)	41.9	101.41	0.82
Stage 3 (n=80)	41.69	119.75	2.81
Stage 4 (n=39)	49.28	147.66	9.7
**CLDN7**	**Median**	**+Error**	**-Error**
Normal (n= 41)	781.07	1180.93	372.16
Stage 1 (n=45)	383.49	651.43	100.56
Stage 2 (n=110)	350.35	702.14	85.61
Stage 3 (n=80)	325.57	607.07	122.29
Stage 4 (n=39)	301.66	654.04	106.76

The transcriptome analysis from TCGA database between the expressions of PIK3CA, SLC6A6, ASAP-1, TMEM-43, CLDN1, and CLDN7 across CRC samples at different stages compared to normal colon (the data was adopted from UALCAN). The median value are represented with respective ±error values.

Table 3. The median values of the expressions of TMEM43, SLC6A6, ASAP-1, PIK3CA, CLDN1 and 7 with the +/- error values in the colon cancer nodal metastasis patients adopted from TCGA database, UALCAN.

The median values for the transcript of signature genes in nodal metastasis of CRC.

**Table 43.**	**Median**	**+Error**	**-Error**
Normal (n=41)	28.14	51.68	18.25
NO (n=166)	42.83	84.42	18.08
N1 (n=70)	48.44	76.95	21.35
N2(n=47)	49.55	74.47	26.25
**SLC6A6**	**Median**	**+Error**	**-Error**
Normal (n=41)	3.13	5.43	1.85
NO (n=166)	22.14	91.15	4.3
N1 (n=70)	31.3	91.95	4.67
N2(n=47)	23.07	88.58	7.7
**ASAP-1**	**Median**	**+Error**	**-Error**
Normal (n=41)	5.37	8.98	2.1
NO (n=166)	8.28	23.84	2.07
N1 (n=70)	9.88	24.8	2.85
N2(n=47)	9.91	22.49	3.21
**PIK3CA**	**Median**	**+Error**	**-Error**
Normal (n=41)	5.79	7.77	3.97
NO (n=166)	5.26	11.35	1.44
N1 (n=70)	5.8	12	2.48
N2(n=47)	6.55	12.5	2.57
**CLDN1**	**Median**	**+Error**	**-Error**
Normal (n=41)	1.37	3.24	0.22
NO (n=166)	42.7	114.58	0.82
N1 (n=70)	41.35	147.66	6.74
N2(n=47)	46.09	107.72	2.81
**CLDN7**	**Median**	**+Error**	**-Error**
Normal (n=41)	788.82	1184.16	372
NO (n=166)	354.12	737.39	85
N1 (n=70)	318.26	607.07	125106
N2(n=47)	291.08	666.19	

The transcriptome analysis from TCGA database between the expressions of PIK3CA, SLC6A6, ASAP-1, TMEM-43, CLDN1, and CLDN7 across CR samples at different nodal metastasis stages (the data was adopted from UALCAN).
